# Haploinsufficiency of the Sec7 Guanine Nucleotide Exchange Factor *Gea1* Impairs Septation in Fission Yeast

**DOI:** 10.1371/journal.pone.0056807

**Published:** 2013-02-15

**Authors:** Alan M. Eckler, Caroline Wilder, Antonio Castanon, Veronica M. Ferris, Rachael A. Lamere, Benjamin A. Perrin, Ross Pearlman, Blaise White, Clifton Byrd, Nicholas Ludvik, Nona Nichols, Kristen Poole-Sumrall, Elizabeth Sztul, Melanie L. Styers

**Affiliations:** 1 Department of Biology, Birmingham-Southern College, Birmingham, Alabama, United States of America; 2 Department of Cell, Developmental, and Integrative Biology, University of Alabama at Birmingham, Birmingham, Alabama, United States of America; University of Cambridge, United Kingdom

## Abstract

Membrane trafficking is essential to eukaryotic life and is controlled by a complex network of proteins that regulate movement of proteins and lipids between organelles. The GBF1/GEA family of Guanine nucleotide Exchange Factors (GEFs) regulates trafficking between the endoplasmic reticulum and Golgi by catalyzing the exchange of GDP for GTP on ADP Ribosylation Factors (Arfs). Activated Arfs recruit coat protein complex 1 (COP-I) to form vesicles that ferry cargo between these organelles. To further explore the function of the GBF1/GEA family, we have characterized a fission yeast mutant lacking one copy of the essential gene *gea1* (*gea1*+/−), the *Schizosaccharomyces pombe* ortholog of *GBF1*. The haploinsufficient *gea1*+/− strain was shown to be sensitive to the GBF1 inhibitor brefeldin A (BFA) and was rescued from BFA sensitivity by gea1p overexpression. No overt defects in localization of arf1p or arf6p were observed in *gea1+/−* cells, but the fission yeast homolog of the COP-I cargo sac1 was mislocalized, consistent with impaired COP-I trafficking. Although Golgi morphology appeared normal, a slight increase in vacuolar size was observed in the *gea1*+/− mutant strain. Importantly, *gea1*+/− cells exhibited dramatic cytokinesis-related defects, including disorganized contractile rings, an increased septation index, and alterations in septum morphology. Septation defects appear to result from altered secretion of enzymes required for septum dynamics, as decreased secretion of eng1p, a β-glucanase required for septum breakdown, was observed in *gea1*+/− cells, and overexpression of eng1p suppressed the increased septation phenotype. These observations implicate *gea1* in regulation of septum breakdown and establish *S. pombe* as a model system to explore GBF1/GEA function in cytokinesis.

## Introduction

Membrane trafficking and protein secretion are essential for maintaining cellular life and underlie many fundamental cellular processes, including cell signaling, nutrient uptake, waste processing, and deposition of the extracellular matrix [1]–[7]. Membrane trafficking collectively refers to the vesicle-mediated movement of proteins and lipids between different cellular membranes [8], [9]. As all membrane and secreted proteins are synthesized in the rough endoplasmic reticulum (ER), proper sorting and transport of these proteins is necessary to ensure that they reach the appropriate destinations for their functions [10]. Hence, cellular life has evolved to develop complex machinery to regulate protein sorting and formation of transport vesicles that carry membrane and secreted proteins throughout the cell.

Vesicle formation within the secretory pathway is regulated by ADP-Ribosylation Factors (Arfs) [11]–[14], small GTPases that oscillate between an active, GTP-bound form and an inactive, GDP-bound form [15]–[17]. Activated Arfs recruit coat proteins to ERGIC (ER-Golgi intermediate compartment), Golgi, and post-Golgi membranes [18]–[22]. These coat proteins drive vesicle formation and promote selection and packaging of the appropriate cargoes into vesicles [23]. Thus, Arf activation drives the formation of transport vesicles that deliver cargo proteins to target membranes. Arf activation is tightly regulated through the action of Guanine nucleotide Exchange Factors (GEFs) and GTPase Activating Proteins (GAPs). GEFs catalyze the exchange of GDP for GTP on Arfs to promote Arf activation [24], [25], whereas GAPs inactivate Arfs through activation of their intrinsic GTPase activity [26], [27].

Arf activation is catalyzed by the Sec7 family of Arf GEFs, named after their founding member *S. cerevisiae SEC7* [28]–[30]. All members of this family possess a highly-conserved Sec7 domain, which catalyzes GDP/GTP exchange on Arfs [30], [31]. Although 9 distinct classes of Sec7 family Arf GEFs have been identified based on phylogeny [28], only two subfamilies have been shown to be inhibited by the fungal metabolite Brefeldin A (BFA), a well-characterized inhibitor of protein secretion [25], [31]–[36]. Of these two families, the GBF1/GEA family has been shown to localize to early secretory compartments, including the ERGIC and Golgi [33], [37], while the SEC7/BIG family has been shown to localize to both secretory and endocytic compartments, including the trans-Golgi Network (TGN) and endosomes [38]–[42].

Characterization of GBF1/GEA family members has provided significant insight into the physiological roles of this family. The GBF1/GEA family has been shown to be required for membrane recruitment of Coat Protein Complex-I (COP-I) in budding yeast and mammalian cells [33], [37], [43], [44]. COP-I facilitates retrograde traffic from the Golgi to the ER, as well as anterograde traffic from the ERGIC to the cis-Golgi [45]–[49]. *H. sapiens* GBF1 has been shown to activate ARF1, ARF4, and ARF5 and to reside in the ERGIC and Golgi [50], [51]. In mammalian cells, siRNA-mediated depletion of GBF1 or expression of the GBF1 dominant-negative mutant E794K results in tubulation or fragmentation of the Golgi and ERGIC and inhibition of protein secretion [33], [38], [50]. GBF1 has also been implicated in post-Golgi trafficking through interactions with the Golgi-localized, gamma-ear-containing, Arf-binding (GGA) coat proteins [52]. In *Saccharomyces cerevisiae*, loss or mutation of the GBF1 family members *gea1* and *gea2* result in defects in ER-Golgi and intra-Golgi transport, alterations in actin morphology, and impaired autophagy [37], [43], [53], [54]. Mutations in the *Drosophila melanogaster* GBF1 homolog *Gartenzwerg* (*Garz*) results in defects in tubulogenesis as a result of impaired formation of polarized epithelia, implicating the GBF1/GEA family in maintenance of cellular polarity [55]. Together, these observations indicate that GBF1 plays important roles in ERGIC and Golgi homeostasis, as well as potential roles in cellular polarization.

In addition to its roles in organellar trafficking, GBF1/GEA family members have also been implicated in regulation of the cell cycle. Depletion of mammalian GBF1 results in cell cycle arrest at the G0/G1 phase and is thought to be associated with the unfolded protein response, ultimately inducing apoptosis [56]. GBF1 activity is also regulated in a cell cycle-specific manner through phosphorylation by the cyclin B-cyclin dependent kinase 1 (CDK1) complex [57]. Loss of *gea1* and *gea2* activity in *S. cerevisiae* causes defects in polarity of the actin cytoskeleton and budding at 37°C, resulting in the formation of multiple buds [53]. However, despite these observations, the precise mechanisms that underlie the role of GBF1/GEA family members in regulation of the cell cycle remain largely unexplored.

The goal of this study was to characterize the function of *gea1*, the fission yeast ortholog of *GBF1*. Because deletion of *S. pombe gea1* gene is lethal, the present study was performed using the haploinsufficient heterozygote strain *gea1*+/−. As predicted based on homology between *S. pombe gea1* and mammalian *GBF1*, we found that the *gea1*+/− strain was sensitive to the GBF1 inhibitor Brefeldin A and that BFA sensitivity could be rescued by overexpression of gea1-YFP. Although no overt defects in Arf localization were detected, we found that a fission yeast homolog of the COP-I cargo sac1 was mislocalized from the ER to the Golgi in *gea1+/−* cells, consistent with impaired COP-I transport. Organellar morphology was generally unaffected in *gea1*+/− cells, but vacuoles appeared slightly enlarged. The most prominent phenotypes in the *gea1*+/− strain were defects in cytokinesis and septation. *Gea1*+/− cells exhibited alterations in contractile ring formation and an increase in the septation index, suggesting a delay in completion of septation. Importantly, the septa exhibited morphological defects, including mislocalization, altered structure, and increased number per cell. Consistent with defects in septation, we observed decreased secretion and mislocalization of eng1p, a β-glucanase involved in septum degradation, in *gea1+/−* cells. Overepression of eng1p suppressed the increased septation phenotype in haploinsufficient cells. Together, our data suggest a role for gea1p in cell-cycle specific secretion of enzymes involved in septation, thus identifying a new function for this family of Arf-GEFs.

## Materials and Methods

### Strains and growth conditions

A list of strains used in this study is shown in Table 1. All strains were derived from the sp286 wild-type strain and the isogenic *gea*+/− strain, which were purchased from Bioneer (Alameda, CA). Cells were cultured in Yeast Extract plus Supplements (YES; MP Biomedical, Solon, OH) or Edinburgh Minimal Media (EMM; MP Biomedical) containing appropriate nutritional supplements. Deletion mutants were selected by growth on YES media containing 200 µg/mL G418. Cells were cultured at 30°C unless otherwise indicated.

**Table 1:** Yeast strains used in this study.

*Strain name*	*Genotype*	*Source*
Wild-type	*ade6*-M210/*ade6*-M216 *ura4*-D18/*ura4*-D18 *leu1*-32/*leu1*-32 *h+/h+*	Bioneer
*gea1*+/−	*gea1*Δ*::kanMX4/gea1 ade6*-M210/*ade6*-M216 *ura4*-D18/*ura4*-D18 *leu1*-32/*leu1*-32 *h+/h+*	Bioneer
Wild-type + gea1-GFP	*ade6*-M210/*ade6*-M216 *ura4*-D18/*ura4*-D18 *leu1*-32/*leu1*-32 *h+/h+ gea1*-GFP::*ura4*	This study
*gea1*+/− + gea1-GFP	*gea1*Δ*::kanMX4/gea1 ade6*-M210/*ade6*-M216 *ura4*-D18/*ura4*-D18 *leu1*-32/*leu1*-32 *h+/h+ gea1*-GFP::*ura4*	This study
Wild-type + eng1-GFP	*ade6*-M210/*ade6*-M216 *ura4*-D18/*ura4*-D18 *leu1*-32/*leu1*-32 *h+/h+ eng1*-GFP::*ura4*	This study
*gea1*+/− + eng1-GFP	*gea1*Δ*::kanMX4/gea1 ade6*-M210/*ade6*-M216 *ura4*-D18/*ura4*-D18 *leu1*-32/*leu1*-32 *h+/h+ eng1-*GFP::*ura4*	This study

### Plasmids and DNA manipulations

The pFA6A-GFP-*ura4MX6* plasmid was a kind gift from Eishi Noguchi (Drexel University College of Medicine, Philadelphia, PA). The pREP4X and pREP4X-eng1 plasmids, which express eng1p under control of the *nmt1*+ promoter, were a kind gift from Carlos Vazquez de Aldana (Universidad de Salamanca, Salamanca, Spain). The pDUAL-YFH1c vector and pDUAL-YFH1c plasmids expressing gea1-YFP, arf1-YFP, arf6-YFP, sac11-YFP (*SPBC19F5.03*), and sac12-YFP (*SPAC3C7.01c*) under control of the full-strength *nmt1+* promoter were purchased from the Riken Bioresource Center DNA Bank (Ibaraki, Japan, deposited by M. Yoshida [58]–[60]).

The polymerase chain reaction (PCR) was used to amplify DNA fragments containing the GFP-*ura4* cassette from pFA6A-GFP-*ura4MX6* as previously described [61], [62]. Primers containing regions of the *gea1* (*gea1*-GFPf and *gea1*-GFPr) and *eng1* (*eng1*-GFPf and *eng1*-GFPr) genes are listed in Table 2. Primers for *eng1* were described previously [63]. PCR reactions contained 1X Phusion® GC Buffer, 1 nM primers, the pFA6A-GFP-*ura4MX6* template, 0.4 mM dNTPs, and Phusion® polymerase (Thermo Fisher Scientific, Inc., Waltham, MA). Reactions were incubated in a Biometra T3 Thermocycler under the following conditions: 1 cycle of 98°C for 1 min; 30 cycles of 98°C for 10 sec, 60°C for 15 sec, and 72°C for 2 min; followed by a final extension at 72°C for 10 min.

**Table 2:** Primers used in this study.

*Primer name*	*Sequence (5′→3′)*
*gea1* forward	TGCCGAAGAGCATGACACTGAGC
*gea1* reverse	CCAACAAGGGCCAGCTTGCGT
*28S* forward	TGAGAAGGGATGTTGGACCTGCTT
28S reverse	ATTGCGTCAACACCACTTTCTGGC
*gea1-*GFPf	GACTTAAATATCAACAACGAAGCCGAAATGAAGAAAGAAAACCTAAAAAATCCCTCACAAACGACTACTGTTCGGATCCCCGGGTTAATTAA
*gea1-*GFPr	CAATGAGCATATATGGAAAAATGATAGTCCCTTTAAATCCATAAAGAATGAGAAAAATTGAAGAGGATAAAAGAATTCGAGCTCGTTTAAAC
*eng1*-GFPf	GCTTGTGGTAATGCGTGCTATGACTCCTCTATATACGGTTGCTCCAATGGTGCACTTGTTGCTGCTCGGATCCCCGGGTTAATTAA
*eng1*-GFPr	TATCCAAAAAGGGTTTCAAGTTGAGAGTAGTTCACGTTCCAGACGTGTATTATGAACAAAATGTAGGAATTCGAGCTCGTTTAAAC
*eng1*cassF	ACTGCAACGGAGCTTGCTAT
CassIntR	GCATCACCTTCACCCTCTCC
CassIntF	TCACCATGCCAAAAATTACACA
*eng1*cassR	AGTCTAAAGGTTCACATCCAGTGT

The aforementioned plasmids and PCR products were transformed into wild-type and *gea1*+/− yeast using the lithium acetate method, as previously described [62]. Transformants were selected on EMM containing appropriate supplements. Transformants expressing eng1-GFP were confirmed by PCR analysis using the primer pairs shown in Table 2 (*eng1*cassF and CassIntR; CassIntF and *eng1*cassR).

### Reverse-transcriptase-PCR (RT-PCR)

RT-PCR was performed to analyze levels of the *gea1* mRNA in wild-type and *gea1+/−* cells. RNA was purified from yeast cells using the RNEasy Mini kit in combination with the RNase-Free DNase set from Qiagen (Valencia, CA). RT-PCR was conducted using the Qiagen OneStep RT-PCR Kit according to the manufacturer's instructions. Briefly, reactions contained 1X RT-PCR buffer, 0.6 µM *gea1*-specific or *28S* rRNA-specific primers (see Table 2), 500 ng total RNA, 0.4 mM dNTPs, 1X Q-solution, and RT-PCR enzyme mix. Reactions were incubated in a Biometra T3 Thermocycler under the following conditions: 1 cycle of 50°C for 35 min; 1 cycle of 95°C for 15 min; 30 cycles of 94°C for 45 sec, 55°C for 45 sec, and 72°C for 1 min; followed by a final extension at 72°C for 10 min. The resulting products were resolved on 0.4% agarose gels and ethidium bromide-stained bands were visualized using a BioDocIt system (UVP, LLC; Upland, CA). Band intensities were quantified using Image J (NIH, Bethesda, MD). The intensities of *gea1* bands were normalized to the corresponding *28S* rRNA bands. Results are reported as percent of the wild-type sample.

### Spot assays

Wild-type cells, *gea1+/−* cells, and *gea1+/−* cells transformed with pDUAL-YFH1c-*gea1* were cultured overnight in YES media, and the density of the resulting cultures was measured by monitoring the absorbance at 600 nm. Equivalent numbers of each cell type were subjected to a 10-fold serial dilution, and 5 µL of each concentration was spotted on plates containing the following media: YES, YES +10 µg/mL Brefeldin A (BFA), YES +0.5 µg/mL FK506, and YES +6 mM valproic acid (VPA). The plates were then incubated at 25°C, 30°C, or 37°C for 3–7 days prior to imaging.

### BFA dose-response assay

To quantify BFA sensitivity of wild-type cells, *gea1+/−* cells, and *gea1+/−* cells transformed with pDUAL-YFH1c-*gea1* or the corresponding empty vector, overnight cultures of each strain were diluted to a final concentration of 5×10^4^ cells/mL in YES media. BFA was added to each culture at the indicated final concentrations ranging from 0–100 µg/mL. The cells were then incubated at 30°C with agitation. After 24 h, the optical density at 600 nm (OD_600_) was analyzed to determine cell number. Results are presented as the percent of control, where the control represents the OD_600_ of each strain in media containing 0 µg/mL BFA.

### Golgi staining with BODIPY® C_5_-ceramide

Wild-type and *gea1+/−* cells from overnight cultures incubated in YES media were resuspended in Hank's Buffered Salt Solution containing 10 mM HEPES, pH 7.4. BODIPY® FL C_5_ ceramide complexed with bovine serum albumin (BSA) or BODIPY® TR C_5_ ceramide complexed with BSA (Invitrogen Molecular Probes, Carlsbad, CA) was added to a final concentration of 5 µM, and the cells were incubated for 30 min at 4°C. The samples were then washed three times with ice-cold medium, followed by incubation in fresh medium at 30°C for 30 min. The samples were then imaged as described below.

### FM4-64 uptake and vacuolar fusion

Overnight cultures of wild-type and *gea1+/−* cells were resuspended in fresh YES media containing 32 µM FM4-64 (Invitrogen Molecular Probes). The cells were then incubated at 30°C for 20 minutes. The cells were then washed and resuspended in fresh YES media and incubated at 30°C for 30 min (for FM4-64 staining) or in dH_2_O for 90 min (to assay vacuolar fusion) as previously described [64], [65]. Following this incubation, the cells were washed once with H_2_O, followed by resuspension in EMM for imaging. Imaging and quantification of vacuolar size was performed as described below.

### Alexa 568-phalloidin/DAPI staining

Alexa 568-phalloidin staining was performed as previously described [66]. Briefly, wild-type and *gea1+/−* cells were fixed by incubating in pre-warmed 4% paraformaldehyde at 30°C for 1 h. The cells were then washed three times in PEM buffer (0.1 M Na Pipes, pH 6.8, 1 mM EGTA, 1 mM MgCl_2_), followed by extraction with 1% Triton X-100 in PEM for 30 s. After 3 additional washes with PEM, the cells were stained with 0.06 units/µL Alexa 568-phalloidin in PEM for 30 min at 37°C. The cells were then washed three times with PEM and resuspended in PEM containing 1 µg/mL DAPI (4′,6-diamidino-2-phenylindole; Sigma Aldrich, St. Louis, MO). Samples were imaged by fluorescence microscopy, as described below.

### Calcofluor white staining

To fix cells for calcofluor staining, cells from overnight cultures in YES media were resuspended in 4% Paraformaldehyde (PFA) in phosphate-buffered saline (PBS) and incubated for 10 min at 30°C. The cells were then washed two times with PBS prior to further staining. Fixed wild-type and *gea1+/−* cells were then resuspended in mounting media (50% glycerol in PBS) containing 50 µg/mL calcofluor white (Sigma-Aldrich; St. Louis, MO) and incubated for 10 min at 30°C. The cells were washed with PBS and resuspended in mounting media prior to imaging and quantification as described below.

### Fluorescence microscopy and quantification

Single color fluorescence images or dual actin/DAPI images were captured using a Zeiss Axioskop 2 fluorescence microscope. Dual color images of YFP and BODIPY® TR C_5_-ceramide were captured using a Perkin Elmer ERS 6FE spinning disk confocal microscope, and images were processed and analyzed in Volocity version 5.3 software (Perkin Elmer, Shelton, CT). Imaging experiments were repeated a minimum of three times using independent cultures. For each experiment, 10–15 images were captured per slide. Both fluorescence and differential interference contrast (DIC) or phase-contrast images were captured for comparison.

For quantification of sac11-YFP and sac12-YFP localization, cells were manually scored as having Golgi localization (punctate distribution throughout the cytoplasm), ER localization (cell cortex and nuclear envelope), or mixed.

Vacuole size was quantified using Image J. Briefly, the pixel area of the largest vacuole in each cell was measured using the software program. The number of cells with vacuolar areas encompassed by each of the stated thresholds was analyzed. The percentage of cells with a given area compared to the total number of cells was then calculated for each threshold and plotted using SigmaPlot (Systat Software, Inc.; San Jose, CA).

For cell size measurements, phase-contrast images were captured from three independent wild-type and *gea1+/−* cultures, as described above. Cell size was analyzed by comparing the length of each cell to that of a micrometer. Results are reported as the percentage of wild-type control cells.

For septum quantification, the number of septated cells was calculated as a percentage of the total number of cells. Each septated cell was scored as normal or abnormal. Cells with abnormal septa included those that contained septa that were not positioned in the center of the cell, those that had septa that were not perpendicular to the length of the cell, those that had misshapen septa, such as forked or abnormally thick septa, and those that contained multiple septa.

To quantify eng1-GFP localization, wild-type and *gea1+/−* cells carrying an integrated GFP cassette at the endogenous *eng1* locus were cultured in EMM media lacking uracil. Images (n  =  30 per culture) were captured and processed using identifical settings. Septated cells were identified on DIC images and were scored based on whether eng1-GFP was visible at the septum.

### Transmission electron microscopy

Wild-type and *gea1+/−* cells were prepared for transmission electron microscopy as described in [67]. Briefly, cells were washed three times with dH_2_O, prior to fixation in 2.5% glutaraldehyde in 0.1 M phosphate buffer, pH 7.2, for 2 h at 4°C. The fixed cells were then washed in 0.1 M phosphate buffer, pH 7.2, prior to post-fixation in 3% potassium permanganate at RT for 90 min. Cells were then washed with distilled water at RT and stained with 2% uranyl acetate in distilled water at 4°C for 30 min. The cells were dehydrated with alcohols in a graduated series and embedded in Epon 812 resin. Ultrathin sections were stained with uranyl acetate and lead citrate and were then observed with an TF-12 Spirit transmission electron microscope (FEI; Hillsboro, Oregon) at an accelerating voltage of 80 kV.

### 1,3-β-Glucanase activity assay

β-glucanase activity was measured as described below in a manner similar to that previously described [68].

#### Collection of media and preparation of cell lysates

Briefly, a volume of overnight cell culture in YES media containing 1×10^9^ wild-type or *gea1+/−* cells was collected and subjected to centrifugation at 1000× *g* for 5 min. The resulting supernatant was collected, and secreted proteins were concentrated using a centrifugal filter unit with a molecular weight cut-off (MWCO) of 30000 Da (Millipore; Billerica, MA), which was subjected to centrifugation at 4000× *g* for 5 minutes at 4°C. The remaining solution was then dialyzed against 50 mM acetate buffer, pH 5.5, using a Slidalyzer dialysis cassette (MWCO, 2000 Da) overnight at 4°C. The volumes of the resulting samples were then equalized. The pelleted cells were resuspended in 50 mM acetate buffer, pH 5.5, and lysed by vortexing in the presence of glass beads (0.5 mm) for 5 minutes at 4°C.

#### 1,3-β-Glucanase Activity Assay

Samples containing secreted proteins and cell lysates were incubated with 0.25% laminarin [β-(1,3)-glucan] in 50 mM acetate buffer, pH 5.5, for 1 h at 30°C. The amount of reducing sugars released by the reaction was then assayed using the Somogyi-Nelson method as previously described [69], [70].

### Quantitative analysis of acid phosphatase secretion

Secretion of acid phosphatase activity was assayed as previously described with slight modifications [67]. Briefly, equal numbers of wild-type and *gea1+/−* cells cultured in YES media were washed and diluted in 10 mL EMM with or without 40 µg/mL BFA and incubated with agitation at 30°C. At 0 h, 1 h, 2 h, 3 h, and 4 h, the OD_600_ of the culture was measured to estimate the number of cells, and a sample was collected for acid phosphatase secretion analysis. The sample was immediately subjected to centrifugation at 25000× *g* for 1 min, and the supernatant, containing secreted acid phosphatase, was stored at 4°C until all samples were collected. Acid phosphatase activity was then assayed by incubating each sample with an equal volume of substrate solution (2 mM *p*-nitrophenyl phosphate, 0.1 M sodium acetate, pH 4.0) for 5 min at 30°C. The reaction was stopped by the addition of a volume of 1M NaOH. Phosphatase activity was then quantified by measuring the absorbance of each reaction at 405 nm. Phosphatase activity (OD_405_) was normalized to cell density (OD_600_) to control for differences in cell growth.

### Western blot analysis

Wild-type and *gea1+/−* cells were harvested from 50 mL cultures by centrifugation and washed with dH2O. The cells were then resuspended in modified TEG buffer (40 mM Tris-HCl, pH 7.5; 1 mM EDTA; 10% glycerol; 0.1% NP-40; 150 mM NaCl) containing 1 mM PMSF and 1X Complete EDTA-free protease inhibitors (Roche; Basel, Switzerland). The cells were lysed by repeated agitation with acid-washed glass beads (Sigma Aldrich). Approximately 50 µg of each cell lysate was then resolved on a 4–20% SDS-PAGE gel (Biorad; Hercules, CA). The proteins were transferred to a polyvinylidene fluoride (PVDF) membrane (Millipore), which was blocked in 5% milk in Tris-buffered saline containing 0.1% Tween-20 (TBST). The membrane was probed with the following primary antibodies: rabbit anti-GFP (Ab290; Abcam; Cambridge, MA; 1∶2000) and mouse anti-β-actin (mAbcam 8224; Abcam; 1∶600). After washing with TBST, the membrane was incubated with Goat anti-mouse horse radish peroxidase (HRP) or goat anti-rabbit HRP secondary antibodies (Sigma, 1∶10000). Bound antibodies were detected using enhanced chemiluminescence Western blotting substrate (Pierce; Rockford, IL). Band intensity was quantified using Image J, and the intensity of the GFP bands was normalized to that of β-actin to control for equal loading.

### Bioinformatic and statistical analyses

Bioinformatic analyses were performed in the Biology Workbench 3.2 (San Diego Supercomputer Center, San Diego, CA). Homologs of *H. sapiens* GBF1 were identified using the BLASTP function, searching relevant databases. Alignments were performed using the CLUSTALW algorithm.

All statistical analyses were performed on a minimum of three independent experiments. Results are reported as mean ± standard deviation (SD) or mean ± standard error of the mean (SEM), as indicated. Significant differences were analyzed using the Student's *t*-test, and *p*-values less than 0.05 were deemed statistically significant.

## Results

### *S. pombe gea1* belongs to the BFA-sensitive GBF1/GEA Arf-GEF family

**Figure 1:**
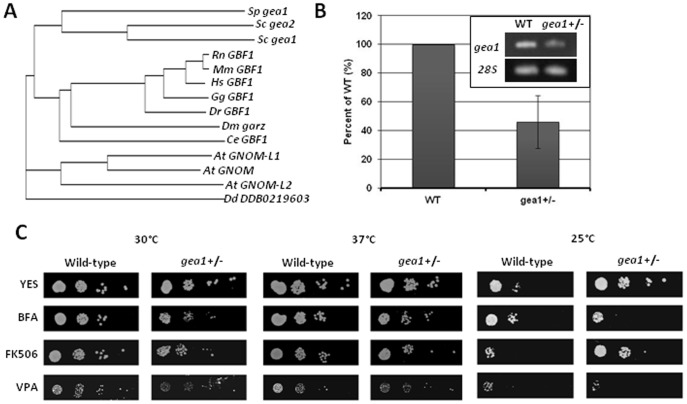
*S. pombe gea1* belongs to the BFA-sensitive GBF1/GEA Arf-GEF family. A. Protein sequences of GBF1 homologs in *Schizosaccharomyces pombe* (*Sp*), *Saccharomyces cerevisiae* (*Sc*), *Rattus norvegicus* (*Rn*), *Mus musculus* (*Mm*), *Homo sapiens* (*Hs*), *Gallus gallus* (*Gg*) *Danio rerio* (*Dr*), *Drosophila melanogaster* (*Dm*), *Caenorhabditis elegans* (*Ce*), *Arabidopsis thaliana* (*At*) and *Dictyostelium discoideum* (*Dd*) were selected based on sequence homology and aligned. B. Total RNA purified from wild-type and *gea1*+/− cells was subjected to RT-PCR using primers specific for *gea1* and the *28S* rRNA (inset). Intensities of the resulting bands were quantified and are presented as the mean ± SD (n = 3). C. Wild-type and *gea1*+/− *S. pombe* cells were subjected to 10-fold serial dilution and spotted on YES media, YES +10 µg/mL brefeldin A (BFA), YES +0.5 µg/mL FK506, or YES +6 mM valproic acid (VPA). Plates were incubated at 30°C, 37°C, or 25°C for 3–5 days. The mutant strain exhibited sensitivity to BFA and FK506 and slight sensitivity to valproic acid.

The GBF1/GEA family of Arf-GEFs is highly conserved, with homologs in all eukaryotes, ranging from plants to fungi to animals (Fig. 1A). Vertebrates, including humans, rats, mice, chickens, and zebrafish, all possess a single ortholog of GBF1. In contrast, the plant *Arabidopsis thaliana* possesses three *GBF1* homologs (*GNOM, GNOM-L1*, and *GNOM-L2*). Unlike *S. cerevisiae*, which possesses two orthologs of *GBF1* (*gea1* and *gea2*), *S. pombe* possesses a single *GBF1* ortholog, similar to vertebrates, flies, and nematodes. Previous studies in budding yeast have shown that *gea1* and *gea2* can functionally compensate for one another [37], complicating functional analyses. Therefore, we chose the fission yeast *Schizosaccharomyces pombe* as a model to characterize GBF1/GEA function.

As complete loss of *gea1* is lethal in *S. pombe*, we used heterozygote *gea1*+/− cells as a model to analyze *gea1* function. To confirm that loss of one copy of *gea1* results in decreased levels of *gea1* mRNA, we performed the reverse transcriptase-polymerase chain reaction (RT-PCR) on RNA purified from wild-type and *gea1+/−* cells using primers specific to *gea1* and the *21S* rRNA (Fig. 1B, inset). Quantification of RT-PCR results and normalization to expression of the *21S* rRNA indicated that expression of the *gea1* mRNA in *gea1+/−* cells was 46% of wild-type (Fig. 1B), demonstrating that loss of one copy of *gea1* was sufficient to induce haploinsufficiency in *gea1* expression.

Our phylogenetic analysis suggests that *gea1* belongs to the GBF1/GEA family of Arf GEFs. Importantly, *H. sapiens* GBF1 was first identified based on the BFA resistance phenotype observed in cells overexpressing GBF1 [51], and the activity of budding yeast gea1p has been shown to be sensitive to BFA [30]. BFA is a fungal metabolite that stabilizes an abortive complex between a subset of Sec7 family GEFs and ARFs [36]. Treatment of cells with BFA blocks protein secretion, causes Golgi fragmentation and accumulation of Golgi proteins in the ER, and leads to cell death at high concentrations [71]. These effects have been shown to be due to Arf-GEF inhibition [36]. Based on our phylogenetic analysis, we hypothesized that *GBF1* and *gea1* are orthologs and that BFA treatment would inhibit gea1p activity, resulting in sensitivity to the drug in *gea1*+/− cells. To test this prediction, wild-type and *gea1*+/− cells were plated on YES medium containing 10 µg/mL BFA and incubated at 30°C, 25°C, or 37°C for 3–5 days prior to imaging. *Gea1*+/− cells exhibited BFA sensitivity at all temperatures analyzed (Fig. 1C), indicating that gea1p is a functional member of the GBF1/GEA family. Interestingly, these results also revealed that *gea1*+/− cells were cold-resistant, suggesting that slower growth at lower temperatures may allow the mutant to overcome growth or cell cycle defects associated with decreased expression of *gea1*.

In addition to BFA sensitivity, we also analyzed the sensitivity of *gea1*+/− cells to the immunosuppressant FK506 and the anti-convulsive agent valproic acid (VPA), drugs previously shown to cause growth sensitivity in a number of *S. pombe* membrane trafficking mutants [67], [72]–[74]. *Gea1*+/− cells exhibited slight sensitivity to FK506 and VPA, although FK506 sensitivity was reversed at 25°C (Fig. 1C). These results are consistent with a defect in membrane transport in *gea1*+/− cells.

**Figure 2:**
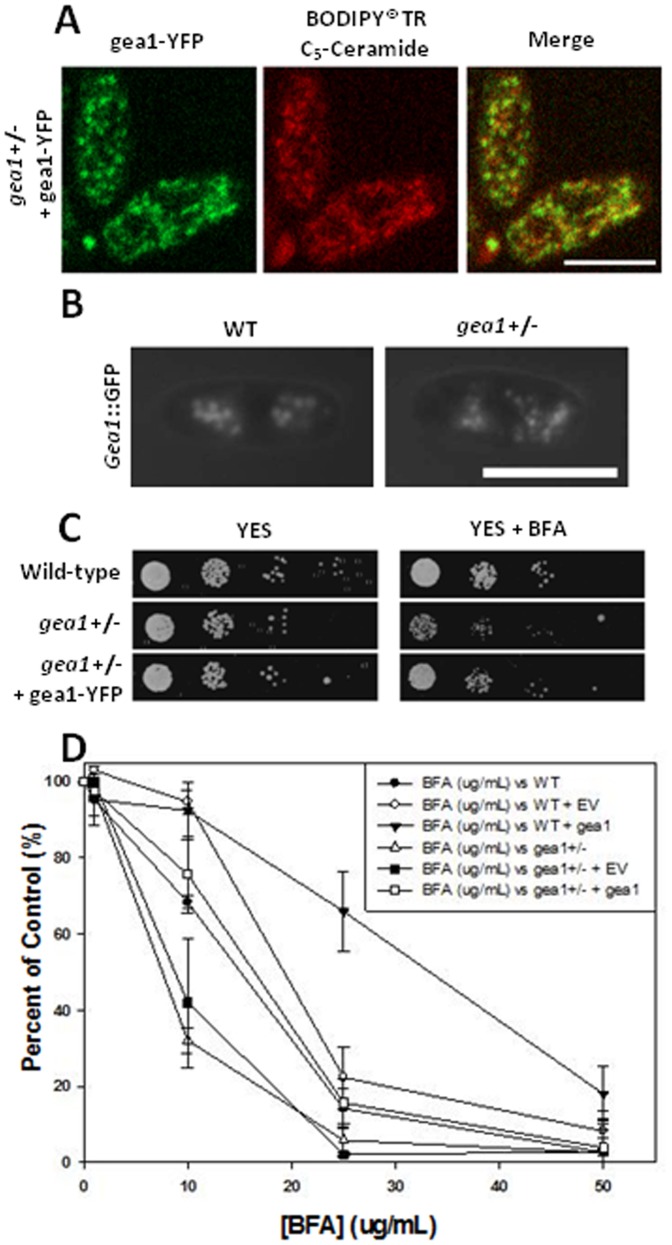
Sensitivity of *gea1+/−* cells to BFA can be rescued by overexpression of gea1p. A. *Gea1+/−* cells were transformed with pDUAL-YFH1c-*gea1* to drive expression of gea1-YFP. Spinning-disc confocal fluorescence microscopy revealed that gea1-YFP localized to punctate structures in the cytoplasm that colocalized with the Golgi-specific stain BODIPY® TR C_5_-ceramide. Scale bar, 14 µM. B. Wild-type and *gea1+/−* cells expressing endogenous gea1 tagged with GFP were imaged by fluorescence microscopy. The gea1-GFP protein localizes to punctate cytoplasmic structures. Scale bar, 10 µM. C. Wild-type, *gea1*+/−, and *gea1+/−* cells transformed with pDUAL-YFH1c-*gea1* (*gea1+/−* + gea1-YFP) cells were subjected to 10-fold serial dilution and spotted on YES media and YES +10 µg/mL BFA. Plates were incubated at 30°C for 3 days. D. Equal numbers (5×10^4^ cells) of wild-type, wild-type (WT) + pDUAL-YFH1c (empty vector, WT + EV), wild-type + pDUAL-YFH1c-*gea1* (WT + gea1), *gea1+/−*, *gea1+/−* + pDUAL-YFH1c, (gea1+/*−* + EV), and *gea1+/−* + pDUAL-YFH1c-*gea1* (gea1+/− + gea1) cells were incubated in YES media containing the indicated concentrations of BFA at 30°C. After 24 h, cell density was assessed by monitoring the optical density of the cultures at 600 nm. The results are reported at as a percentage of the density of control untreated cultures. Error bars represent mean ± SE (n = 3). Restoration of gea1p expression suppressed the BFA sensitivity of *gea1+/−* cells.

To confirm that the BFA sensitivity phenotype was due to decreased expression of gea1p, we created a “rescued” *gea1+/−* strain by transforming the strain with a vector driving expression of gea1-YFP. Examination of *gea1+/−* cells with low level overexpression of gea1-YFP by confocal fluorescence microscopy revealed that gea1-YFP was found in small punctate dots in the cytoplasm that colocalized with the Golgi-specific dye BODIPY® TR C_5_-ceramide (Fig. 2A; [75], [76]). Some aggregation was observed with higher expression (data not shown). Punctate cytoplasmic localization was also observed for gea1-GFP under control of its endogenous promoter (Figure 2B). This localization pattern is consistent with that observed for the human GBF1 protein, which also localizes to the Golgi [33]. Importantly, overexpression of gea1p-YFP restored the growth of *gea1+/−* cells in the presence of BFA, both in a spot assay (Fig. 2C) and in a quantitative dose-response assay (Fig. 2D). Consistent with this observation, overexpression of gea1-YFP in the wild-type strain resulted in BFA resistance (Fig. 2D), similar to previous studies of GBF1 in mammalian cells [51]. These results confirm that BFA sensitivity of *gea1+/−* cells is indeed due to loss of gea1p expression and establish gea1p as a homolog of the mammalian GBF1 protein.

### *Gea1* haploinsufficiency affects COP-I-dependent transport

**Figure 3:**
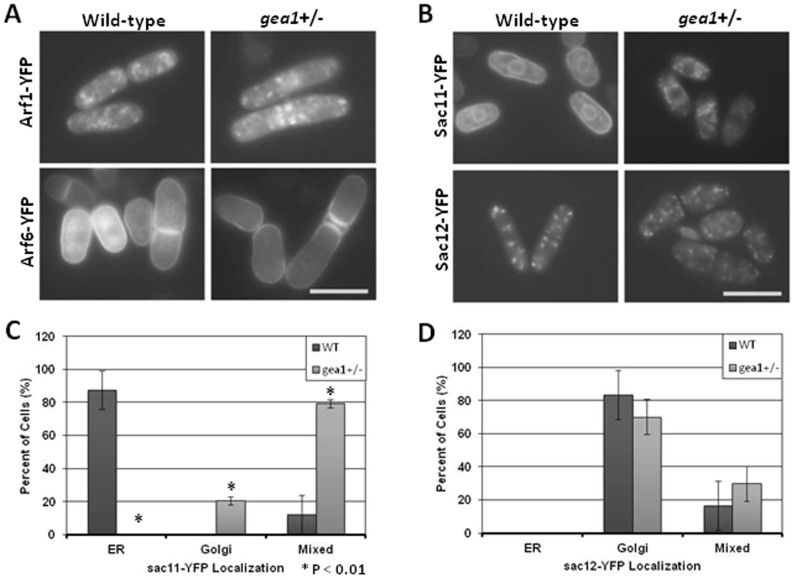
COP-I-dependent transport is impaired in *gea1+/−* cells. A. Wild-type and *gea1+/−* cells were transformed with pDUAL-YFH1c-*arf1* or pDUAL-YFH1c-*arf6* and imaged by fluorescence microscopy. Scale bar, 10 µM. B. Wild-type and *gea1+/−* cells were transformed with pDUAL-YFH1c-*sac11* (SPBC19F5.03) or pDUAL-YFH1c-*sac12* (SPAC3C7.01c) and imaged by fluorescence microscopy. Scale bar, 10 µM. C. Localization of sac11-YFP in wild-type (n = 207) and *gea1+/−* cells (n = 110) was scored as ER (surrounding the cell cortex and nuclear envelope), Golgi (punctate in the cytoplasm), or mixed. Sac11-YFP was predominately found in the ER in wild-type cells and in the Golgi and mixed in *gea1+/−* cells. D. Localization of sac12-YFP in wild-type (n = 204) and *gea1+/−* cells (n = 147) was scored as ER, Golgi, or mixed. Sac11-YFP was predominately found in the Golgi in both wild-type and *gea1+/−* cells. Error bars represent the mean ± SD from 3 independent experiments.

The GBF1/GEA family of Arf-GEFs has previously been shown to regulate COP-I-mediated trafficking through activation of Arfs [33], [37], [43], [51]. Therefore, we analyzed Arf localization in *S. pombe*. Fission yeast possess two arf homologs, termed *arf1* and *arf6*. Sequence analysis has revealed *arf1* to belong to the class I/II family of Arfs, which localize to the Golgi and endosomes and exhibit BFA-sensitive activation [33], [77]. In contrast, *arf6* belongs to the class III Arfs, which localize to the plasma membrane and are not sensitive to BFA [77], [78]. Wild-type and *gea1+/−* cells were transformed with plasmids overexpressing YFP-arf1p or YFP-arf6p. In wild-type cells, Arf1p localized to punctate structures in the cytoplasm (Fig. 3A). These structures exhibited slight overlap with the Golgi-specific dye BODIPY® TR C_5_-ceramide (Supplemental Figure S1A, arrowheads). Limited colocalization with the Golgi was not unexpected, as class I/II arfs have also been shown to be recruited to the endosomes and trans-Golgi network by the Sec7/BIG family of Arf-GEFs [38], [79]–[81]. In contrast, arf6p localized to the cell surface and the membranes surrounding the septum. No overt changes were observed in arf1p or arf6p localization, although septum structure appeared altered in the *gea1*+/− cells. However, subtle changes in arf localization, such as partial changes in distribution between the cytoplasm and membrane compartments, could not be excluded.

GBF1 activity is required for recruitment of COP-I to Golgi membranes [33], [51]. Therefore, we next tested whether COP-I-dependent trafficking pathways were operational in *gea1*+/− cells by analyzing localization of sac1 homologs. Sac1 is a lipid phosphatase that exhibits specificity for phosphatidylinositol-4-phosphate (PI4P) [49] COP-I has been shown to be required for its retention in the ER, and human sac1 mutants incapable of binding COP-I accumulate in the Golgi due to inhibition of retrograde transport [49]. Sequence analysis revealed two potential homologs for *S. pombe sac1*, *SPBC19F5.03* and *SPAC3C7.01c*, which we termed *sac11* and *sac12*, respectively. Overexpression of sac11p-YFP in wild-type cells revealed that sac11p was found at the cell cortex and surrounding the nucleus, consistent with ER localization (Fig. 3B). In contrast, sac12p-YFP localized to punctate spots that colocalized with a Golgi marker in wild-type cells (Fig. 3B and Supplemental Figure S1B). These observations suggest that sac11p is orthologous to the COP-I cargo, mammalian sac1. In *gea1+/−* cells sac11-YFP was found in punctate structures that colocalized with the Golgi marker BODIPY® TR C_5_-ceramide, while Sac12p localization was unchanged (Fig. 3B and Supplemental Figure S1B). Quantification of these results revealed that sac11-YFP, but not sac12-YFP, was mislocalized in *gea1+/−* cells (Fig. 3C,D). These data suggest that recycling of sac11p from the ER to the Golgi is selectively impaired in *gea1+/−* cells, consistent with impaired COP-I activity.

### Organellar morphology is largely unaffected in *gea1+/−* cells

**Figure 4:**
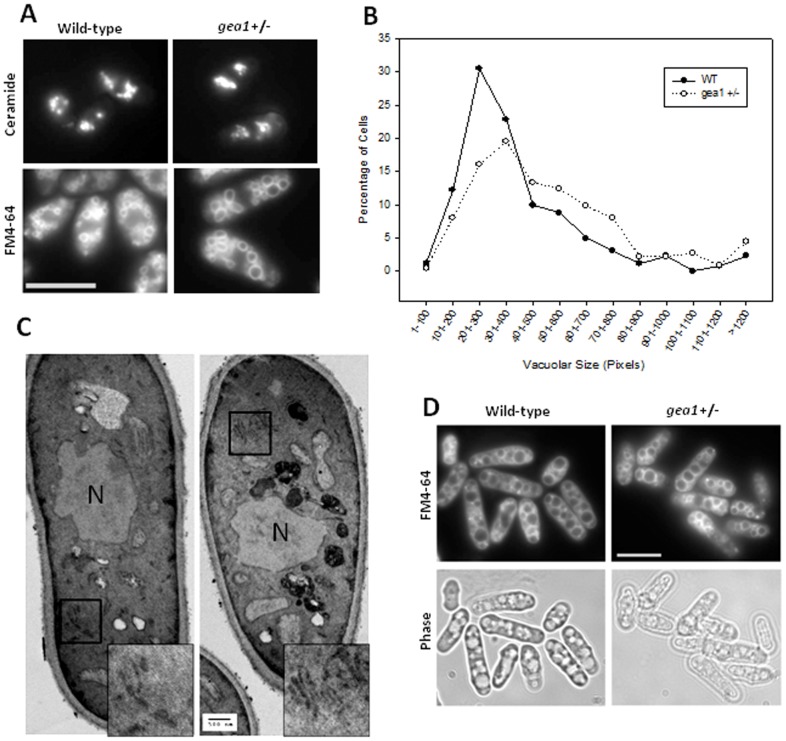
Organellar morphology in *gea1+/−* cells. A. Wild-type and *gea1+/−* cells were stained with 5 µM BODIPY FL C_5_-ceramide to label the Golgi or with 32 µM FM4-64 to label the vacuole. Staining was visualized by fluorescence microscopy. Scale bar, 10 µM. B. The pixel area associated with the largest vacuole was measured using Image J for individual wild-type (n = 262) and *gea1+/−* (n = 225) cells. The percentage of cells with vacuolar sizes of the indicated ranges was plotted using SigmaPlot. C. Wild-type (A) and *gea1*+/− cells (B) were subjected to transmission electron microscopy to visualize membranous structures. Representative images show flat ribbon-like structures consistent with Golgi membranes (insets) that appear similar in wild-type and *gea1+/−* cells. N, nucleus. Scale bar, 500 nm. D. Cells were labeled with FM4-64, followed by incubation in H_2_O for 90 min to induce vacuolar fusion. Scale bar, 10 µM.

Pharmacological or siRNA-mediated inhibition of GBF1 activity in mammalian cells results in fragmentation and tubulation of the Golgi [33], [50]. Therefore, we analyzed the morphology of secretory and endocytic organelles in *gea1+/−* cells. Wild-type and *gea1+/−* cells were stained with the Golgi-specific dye BODIPY FL C_5_-ceramide [75], [82]. Ceramide staining revealed no overt differences in the punctate Golgi structures (Fig. 4A and Supplemental Figure S1). Results from transmission electron microscopy also suggested that Golgi membranes remain intact in *gea1+/−* cells (Fig. 4C).

Morphology of vacuoles and other endocytic organelles was assessed by uptake of the fluorescent dye FM4-64. Importantly, uptake of FM4-64 is dependent upon a competent endocytic pathway [64], and both wild-type and *gea1+/−* cells successfully took up the dye, indicating that endocytosis was not inhibited by loss of *gea1*. However, analysis of vacuoles stained with FM4-64 revealed a slight increase in vacuolar size (Fig. 4A). To quantify this increase in vacuolar size, the pixel area associated with the largest vacuole in each cell was analyzed. This analysis revealed a trend towards larger vacuoles in *gea1+/−* cells when compared to wild-type cells (Fig. 4B). We next analyzed whether increased vacuolar size in *gea1+/−* cells was associated with changes in vacuolar fusion. Incubation of fission yeast in a hypotonic solution results in an increase in vacuolar fusion [65]. Therefore, we stained wild-type and *gea1+/−* cells with FM4-64, followed by incubation in H_2_O for 90 minutes to induce fusion. No changes in vacuolar fusion were observed in *gea1+/−* cells compared to wild-type cells, suggesting that the enlarged vacuoles were competent for fusion (Fig. 4D).

### *Gea1+/−* cells exhibit defects in cytokinesis and septation

**Figure 5:**
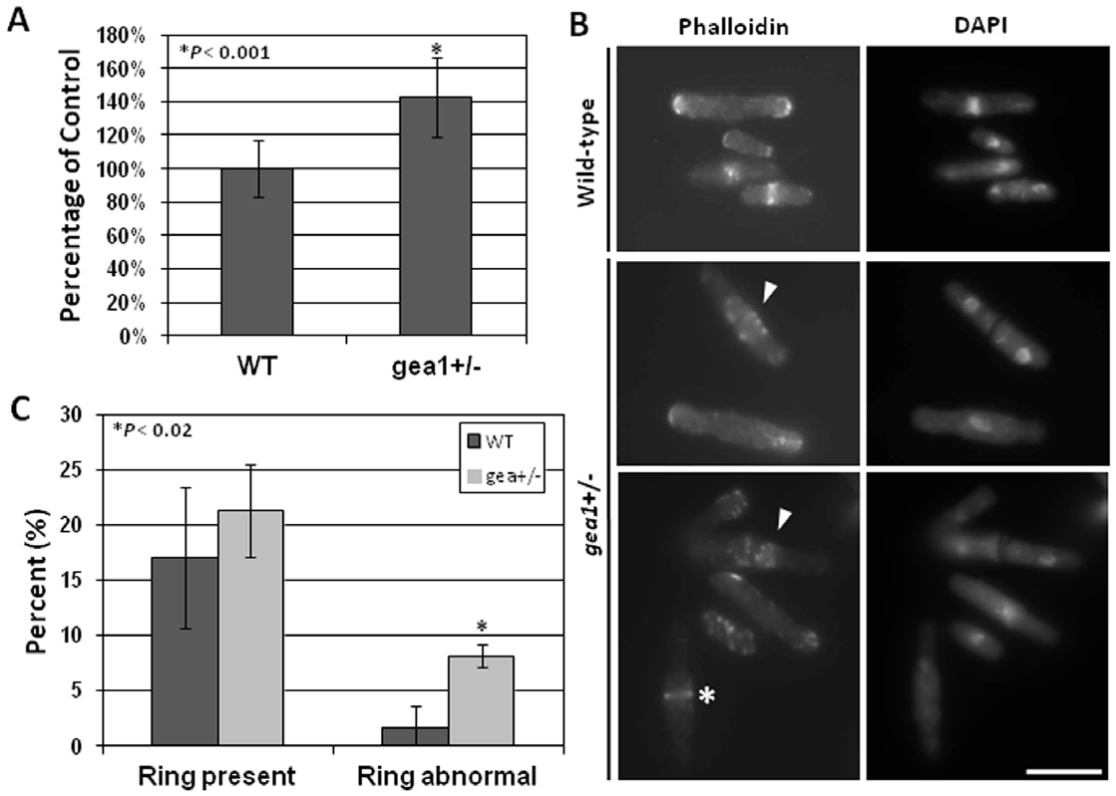
*Gea1+/−* cells exhibit defects in cytokinesis. A. Cell length of wild-type (n = 221) and *gea1+/−* cells (n = 205) was measured based on phase-contrast images. Error bars represent mean ± SEM from 3 independent experiments. *p<0.001. B. Wild-type and *gea1*+/− cells were stained with DAPI to visualize nuclei and Alexa 568-phalloidin to visualize F-actin. Staining in *gea1*+/− cells revealed the presence of numerous septated cells exhibiting disorganized contractile acto-myosin rings. Asterisk indicates a normal contractile ring, and arrowheads indicate abnormal rings in *gea1+/−* cells. Scale bar, 10 µM. C. Quantification of the percentage of cells containing an actomyosin ring or an abnormal ring in wild-type (n = 176) and *gea1*+/− cells (n = 180). Error bars represent mean ± SD from 3 independent experiments. *p<0.02.

Microscopic analysis of *gea1*+/− cells revealed a large number of septated cells and potential defects in septum structure (see Fig. 3A). Based on these observations, we hypothesized that *gea1*+/− cells have defects in the cell cycle, likely in assembly of the contractile actomyosin ring and/or septum. Cell cycle delays in *S. pombe* are associated with an increase in cellular length [83]. Therefore, to assess whether the cell cycle might be altered in *gea1+/−* cells, we measured the length of wild-type and *gea1+/−* cells. The mutant cells were approximately 40% longer than the wild-type cells, consistent with a cell cycle delay (Fig. 5A).

To determine whether the cell cycle delay was associated with impaired cytokinesis, we stained wild-type and *gea1*+/− cells with Alexa 568-phalloidin, which specifically binds to F-actin, labeling actin patches and actomyosin rings [84]. In wild-type cells, actin staining was clearly visible in contractile rings located in the medial portion of binucleate cells and at the cell tips (Fig. 5B). Actin patches were also visible at the tips of *gea1*+/− cells, although they appeared slightly less organized than in the wild-type cells. However, the most notable difference between the two strains was in the medial contractile ring structures in the *gea1+/−* cells. Although some normal contractile rings were present (Figure 5B, asterisk), many of the contractile rings appeared to be comprised of extended networks of medial actin patches, in some cases surrounding an abnormal septum (Figure 5B, arrowheads). Quantification of these results revealed that although the numbers of medial actin ring structures were similar in wild-type and *gea1+/−* cells, the haploinsufficient cells exhibited a significant increase in the number of disorganized, abnormal rings compared to the wild-type cells (Figure 5C).

**Figure 6:**
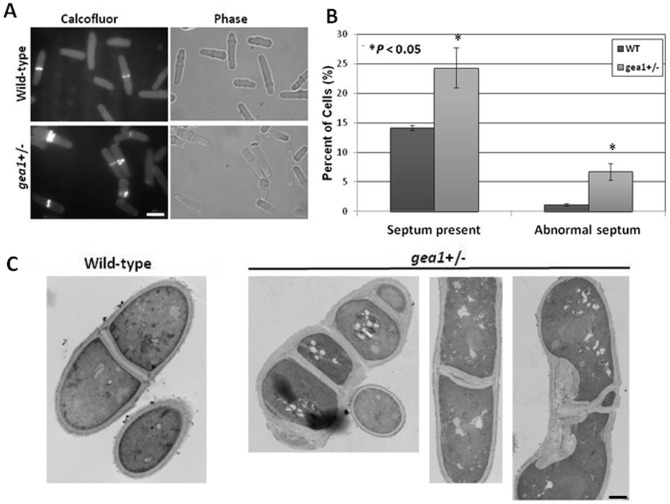
*Gea1+/−* cells exhibit alterations in septum number and morphology. A. Wild-type and *gea1*+/− cells were stained with calcofluor white to visualize septa and imaged by fluorescence microscopy. Scale bar, 10 µM. B. Quantification of A. Cells were scored as having abnormal septa if multiple septa were present, or if the septum was mislocalized, abnormally thick, or forked. Error bars represent mean ± SD from 3 independent experiments. C. Wild-type and *gea1*+/− cells were subjected to transmission electron microscopy to visualize septum defects. Representative images of multi-septated cells and septa with morphological abnormalities are shown. Scale bar, 1 µm.

Defects in actomyosin ring assembly led us to analyze septum morphology in *gea1*+/− cells. In *S. pombe*, the septum is a cell wall-related structure that forms between cells undergoing cytokinesis and is subsequently degraded to separate the two new cells [85]–[87]. Staining with calcofluor revealed that *gea1*+/− cells exhibit a significantly higher septation index and have an increased number of abnormal septa (Fig. 6B). Morphological abnormalities observed in septated cells included the presence of multiple septa per cell and/or septa that were forked, mislocalized, or abnormally thick (Fig. 6A). Ultrastructural observation of these cells by transmission electron microscopy confirmed the presence of altered septum structures (Fig. 6C).

### Secretion of eng1p is selectively impaired in *gea1+/−* cells

**Figure 7:**
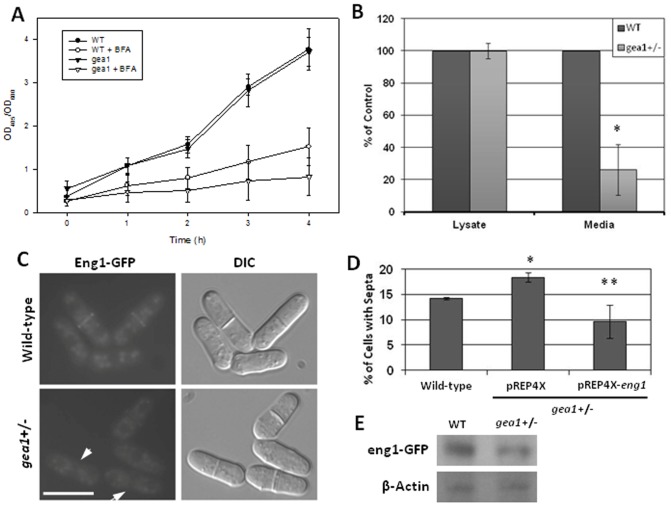
Secretion of the β-glucanase eng1p is selectively inhibited in *gea1+/−* cells. A. Equal numbers of wild-type and *gea1*+/− *S. pombe* cells were used to inoculate 10 mL of EMM media. At the indicated times, an aliquot of the media was subjected to spectrophotometric determination of secreted acid phosphatase activity by monitoring cleavage of the substrate *p*-nitrophenyl phosphate at 405 nm. Acid phosphatase activity was normalized to cell density, determined by monitoring absorbance at 600 nm (OD_600_). Treatment with 40 µg/mL BFA to inhibit secretion served as a negative control. B. β-glucanase activity in cell lysates and in media was measured by quantifying release of reducing sugars from the substrate laminarin as previously described [68]. Normal levels of β-glucanase activity were detected in wild-type and *gea1*+/− cell lysates, but β-glucanase activity was decreased in the media from *gea1*+/− cells. Error bars represent the mean ± SD from 3 independent experiments. *p<0.02 C. Wild-type and *gea1+/−* cells expressing eng1p tagged with GFP under control of the endogenous promoter were imaged by fluorescence microscopy. Localization of eng1-GFP to the septum was substantially decreased in *gea1+/−* cells. Arrows indicate septated cells with no visible eng1-GFP at the septum. Scale bar, 10 µM. D. *Gea1*+/− cells were transformed with either the empty pREP4X vector or pREP4X carrying *eng1*. Wild-type and both *gea1+/−* strains were then stained with calcofluor, and the percentage of cells with septa were counted for each culture. Overexpression of eng1p rescued the excessive septation defect of *gea1+/−* cells. Error bars represent the mean ± SD from 3 independent experiments. *p<0.02 compared to wild-type. ***p<0.05 compared to *gea+/− +* pREP4X. E. Lysates from wild-type and *gea1*+/− cells in which endogenous eng1 was tagged with GFP (shown in C) were subjected to immunoblot analysis using antibodies directed against GFP and β-actin. No significant difference in eng1p-GFP levels was observed between the wild-type and *gea1+/−* cells (*p* = 0.70).

Production and degradation of the septum require intact membrane transport pathways [88]–[93], and enzymes that make and degrade the septum are secreted in a cell cycle-regulated manner [94], [95]. These observations suggest a potential role for gea1p in transport of these enzymes to the septum. To determine whether defects in septation were due to generalized inhibition of secretion in *gea1+/−* cells, we analyzed secretion of acid phosphatase, a highly secreted protein in fission yeast [96]. Levels of acid phosphatase secretion were similar between wild-type and *gea1+/−* cells (Fig. 7A), indicating that general secretion was not impaired by loss of one copy of *gea1*. Treatment of wild-type cells with 40 µg/mL BFA, a dose previously shown to inhibit secretion in *S. pombe* [97], resulted in substantial inhibition of acid phosphatase secretion, which was even greater in *gea1+/−* cells (Fig. 7A). Although the BFA hypersensitivity phenotype suggests that general protein secretion may be partially regulated by *gea1*, the single remaining copy of *gea1* is clearly sufficient to support secretion of at least some proteins in *gea1+/−* cells.

As we observed no defects in general protein secretion in *gea1+/−* cells, we next tested whether specific defects in transport of enzymes relevant to septation were present in *gea1+/−* cells. We chose eng1p, a β-glucanase involved in degradation of the primary septum, as a model enzyme based on previous evidence that eng1p is secreted, requiring the function of the exosome complex and *rho4* [63], [89], [91]. Loss or altered secretion of eng1p results in increases in the septation index and in the number of septa per cell [63], [89], [91], similar to the morphological defects we observed in *gea1+/−* cells. Importantly, secretion of eng1p differs significantly from that of acid phosphatase because eng1p is both produced and secreted in a cell cycle-specific manner [63], [89], [91]. To analyze eng1p secretion in *gea1*+/− cells, we performed a β-glucanase activity assay, which has previously been established as a highly specific measure of β-glucanase levels [63]. Eng1p activity levels were similar in lysates from wild-type and *gea1*+/− cells, indicating similar levels of expression (Fig. 7B). However, substantially less eng1p activity was observed in the medium from *gea1*+/− cells (Fig. 7B), suggesting a defect in secretion. To assess whether decreased secretion of eng1p was accompanied by mislocalization of the protein, we tagged endogenous *eng1* with GFP. Importantly, Western blot analysis revealed no significant difference in eng1-GFP expression between the wild-type and *gea1+/−* strains (*p* = 0.70), consistent with enzymatic assays (compare Fig. 7A and 7E). As previously described, eng1-GFP localized to the septum in wild-type cells ([63] and Fig. 7C). A total of 94% of septated wild-type cells exhibited localization of eng1-GFP to the septum. In contrast, in *gea1+/−* cells, levels of eng1p were decreased or absent from the septa (Fig. 7C), with only 59% of septated *gea1+/−* cells having detectable levels of eng1-GFP at the septum. These results indicate that loss of one copy of *gea1* is sufficient to impair secretion of specific cargo proteins required for cytokinesis. Importantly, overexpression of eng1p rescued the excessive septation defect in the *gea1+/−* strain (Figure 7D), indicating that defects in cytokinesis and septation in *gea1+/−* cells are likely due to impaired cell cycle-specific secretion of enzymes, such as eng1p, that are required for septation.

## Discussion

In this study, we have established *S. pombe* as a model system to analyze Arf-GEF function. Unlike *S. cerevisiae, S. pombe* possesses only one ortholog of *GBF1*, similar to vertebrates (Fig. 1A). Importantly, we have shown that *gea1+/−* cells exhibit sensitivity to the GBF1 inhibitor BFA (Fig. 1C, 2C, 2D) and that overexpression of gea1-YFP results in BFA resistance (Fig. 2D), confirming that *gea1* is a member of the GBF1/GEA family of Arf GEFs. Additionally, a fission yeast homolog of the COP-I cargo sac1 was mislocalized from the ER to the Golgi in *gea1+/−* cells, consistent with a role for *gea1* in COP-I-dependent transport, similar to its mammalian counterpart [33], [49].

Comparisons with data from other fission yeast membrane trafficking mutants are consistent with a role for *gea1* in membrane transport. *Gea1*+/− cells exhibit slight sensitivity to FK506 and valproic acid (Fig. 1C), similar to strains with mutations or deletions in the subunits of the AP-1 coat complex, and the GTPases *rho3*, *ypt3*, and *ryh1* [67], [72]–[74]. However, although *gea1* mutants exhibit some phenotypic overlap with these mutants with respect to FK506 and VPA sensitivity and defects in septation, some of the phenotypic aspects associated with *gea1* haploinsufficiency appear to be quite different. For example, we found that vacuolar size was slightly increased and secretion of acid phosphatase was not impaired in *gea1+/−* cells (Fig. 4B, 7A). In contrast, deletion of the *apm1* subunit of AP-1 was shown to result in increased vacuolar fragmentation and decreased acid phosphatase secretion [67]. We also found that *gea1* haploinsufficiency resulted in cold resistance and that FK506 sensitivity could be reversed at lower temperatures (Fig. 1C), differing from previous studies of other membrane trafficking mutants [67], [72]–[74]. FK506 is an inhibitor of calcineurin, and inhibition or loss of calcineurin activity has been shown to impair both septation and membrane transport in fission yeast [67], [72]–[74], [98]. We speculate that slower growth at colder temperatures may allow sufficient time for delayed trafficking of enzymes required for septation in haploinsufficient *gea1+/−* cells, potentially underlying reversed sensitivity to FK506. Together, these observations suggest that, although *gea1* is appears to be important for membrane trafficking and septation in *S. pombe*, this role may be distinct from that of previously characterized membrane trafficking mutants in fission yeast.

As mentioned previously, we observed no defects in secretion of acid phosphatase in *gea1+/−* cells (Fig. 7A), despite the observation that treatment with BFA has been shown to block protein secretion in fission yeast ([97] and Fig. 7A). However, previous studies have shown that impaired function of GBF1/GEA family members in both mammalian cells and budding yeast causes cargo-specific defects in protein secretion. Secretion of transmembrane, but not soluble, cargoes is decreased in mammalian cells depleted of GBF1 [50], and secretion of only a subset of cargoes is decreased in budding yeast cells with mutations in *gea1* [37]. Together, these results suggest that the GBF1/GEA family may selectively regulate secretion of specific cargo proteins, as opposed to total protein secretion.

In addition to impaired secretion of transmembrane cargo proteins, >90% depletion of GBF1 in mammalian cells has also been shown to result in tubulation and fragmentation of the Golgi and decreased recruitment of Arf to the membrane [50]. However, in the *gea1+/−* model, no overt defects in Golgi structure or Arf membrane recruitment were observed (Fig. 3A, 4A, 4C). Several differences between *S. pombe gea1+/−* cells and mammalian GBF1-depleted cells may underlie these inconsistencies. First, *gea1+/−* cells retain 50% of *gea1* expression (Fig. 1B), which may be sufficient to perform the normal housekeeping activities of this protein in fission yeast. Second, the Golgi architecture differs substantially between yeast and mammalian cells. In fission yeast, the Golgi is present as multiple polarized mini-stacks, in contrast to the single Golgi ribbon present in mammalian cells [99]. This “fragmented” nature may reflect differences in Golgi biogenesis between yeast and mammalian cells and may also hinder observations of subtle changes in Golgi structure or Arf recruitment. Third, fission yeast have only one class I/II Arf (arf1p, Fig. 3A), but they possess three Arf-GEFs predicted to localize to the Golgi and/or endosomes, gea1p, sec71p, and sec72p. In contrast, the mammalian homologs of these Arf GEFs (GBF1, BIG1, and BIG2) exhibit distinct specificities for the four Golgi-localized Arfs present in mammalian cells [50], [51], [100]. Therefore, there may be functional redundancies between some of the fission yeast Arf-GEFs that are not present in mammalian cells with respect to maintenance of Golgi structure, Arf recruitment, and general secretion.

Analysis of the *gea1+/−* mutant has uncovered a novel role for *gea1* in regulation of vacuolar size. Consistent with the localization of arf1p to both the Golgi and the vacuole, we observed a slight increase in vacuolar size in *gea1+/−* cells (Fig. 4A, B). Interestingly, mutations in *S. cerevisiae gea1* and *gea2* have previously been shown to cause a slight increase in vacuolar fragmentation, consistent with a role for this family of proteins in vacuolar homeostasis in yeast [37]. However, the precise mechanisms underlying this phenotype remain unclear. Alterations in trafficking of ion channels to vacuolar membranes could alter the osmotic pressure of the vacuole, resulting in swollen vacuoles. Alternatively, improper delivery of factors required for vacuolar fission and fusion could dysregulate the precise balance between fission and fusion required to maintain vacuolar size.

The major defects observed in *gea1+/−* cells were associated with cytokinesis and septation. We observed disorganization of contractile actomyosin ring (CAR) structures (Fig. 5) and cells with multiple septa, mislocalized septa, and malformed septa (Fig. 6). Cytokinesis is a tightly regulated process in *S. pombe*, governed by signaling of kinases of the septation initiation network [85], [101]. The septum forms just behind the CAR, and septum formation is tightly linked to CAR assembly [102]. Septum formation requires polarized delivery of glucan synthases, such as bgs1p, that synthesize the new cell wall material [103], [104] and glucanases, such as eng1p and agn1p, that rapidly break down the septum to separate the two new daughter cells [63], [94]. Defects in delivery of these enzymes to the site of septum assembly and breakdown result in impaired cell division, similar to that observed in *gea1+/−* cells (Fig. 6). As an initial approach to determine whether secretion of cell cycle-specific enzymes was impaired in *gea1+/−* cells, we chose to examine eng1p secretion. Eng1p has been shown to be required for dissolution of the primary septum, and defects in secretion of eng1p have been shown to affect septation in fission yeast [63], [89], [91]. Specifically, mutation of *rho4* and genes belonging to the exocyst complex, a part of the machinery required for vesicle fusion, have been shown to decrease secretion of eng1p, resulting in impaired septum breakdown and septation defects overlapping those we observed in *gea1+/−* cells [89], [91]. Furthermore, the cell cycle-specific expression pattern of eng1p has been well-characterized and is demonstrated to peak specifically during septation [63]. Our results demonstrate that secretion of eng1p is impaired in *gea1+/−* cells. Although eng1 activity was similar in cell lysates from wild-type and *gea1+/−* cells, secretion of eng1p activity from *gea1+/−* cells was approximately 20% of the wild-type (Fig. 7B). We also observed a substantial decrease in localization of eng1-GFP to septa in *gea1+/−* cells. Furthermore, similar to *rho4*Δ mutants [89], overexpression of eng1p was able to suppress the excessive septation defect in *gea1*+/− cells (Fig. 7D). These results suggest that, although eng1p is produced, only a small fraction of eng1p reaches the septum during cytokinesis. We hypothesize that the mislocalized eng1p is likely degraded due to lack of export.

Characterization of *gea1* function in *S. pombe* may provide insight into a conserved network of proteins that connect membrane traffic and cytokinesis in other eukaryotes. In the present study, we observed defects in trafficking of sac11p in *gea1+/−* cells (Fig. 3B, C). Importantly, the *S. cerevisiae* homolog of sac1p has been shown to play a role in septation, suggesting that this pathway may be conserved in yeast [105]. The secretory pathway also appears to play an important role in cytokinesis in higher eukaryotes. In *C. elegans*, treatment of embryos with Brefeldin A results in regression of the cleavage furrow, suggesting that secretion is required for completion of cytokinesis [106]. Secretion also occurs at the cleavage furrow in sea urchin embryos late in cytokinesis and is independent of constriction of the cleavage furrow [107].

Characterizing the network of proteins that work with *gea1* to drive septation in fission yeast may also shed light on these pathways in mammalian cells. For example, defects in contractile actomyosin ring assembly and septum structure similar to those found in the *gea1+/−* mutant have been observed in cells expressing mutants of pik1p, a fission yeast phosphatidylinositol-4-kinase (PI4K) [108]. These overlapping phenotypes suggest that *gea1* and *pik1* may act in the same pathway to control cytokinesis and septum biogenesis. In mammalian cells, PI4K type IIIα has been shown to be required for Rab1-mediated recruitment of GBF1 to Golgi membranes [109]. Additionally, mammalian PI4KIIIβ has been shown to colocalize with GBF1 during hepatitis c virus replication and, like GBF1, to be required for viral replication [110]. Together these results suggest that protein networks connecting GBF1/GEA family members and PI4K family members may be conserved in higher eukaryotes.

Together, our observations suggest a novel role for *gea1* in polarized, cell cycle-specific secretion. The selective inhibition of polarized secretion of eng1p to the septum suggests that the GBF1/GEA family may play previously unappreciated roles in cell cycle progression. GBF1 has previously been implicated in regulation of the cell cycle, as depletion of GBF1 results in cell cycle arrest at the G0/G1 phase and is associated with induction of ER unfolded protein response, ultimately inducing apoptosis [56]. GBF1 activity has also been shown to be regulated in a cell cycle-specific manner through phosphorylation by the cyclin B-cyclin dependent kinase 1 (CDK1) complex [57]. In budding yeast and in *Drosophila*, loss of *gea1/2* and *garz* activity have been associated with polarity defects, suggesting a role in polarized secretion [53], [55], [111]. Therefore, future studies to examine the secretion of other enzymes required for septum formation and of polarity factors required for CAR positioning in *gea1+/−* cells may help to identify a network of interactions required for proper cell cycle progression and polarity determination in fission yeast and other organisms.

In summary, our data supports the hypothesis that gea1p plays an important role in cytokinesis in *S. pombe* by regulating the trafficking of key components required for the septation. These studies shed light on a novel role for the GBF1/GEA family of Arf-GEFs and establish *S. pombe* as a model to explore GBF1/GEA function.

## Supporting Information

Figure S1 **Analysis of Golgi localization.** A. Wild-type and *gea1+/−* cells were transformed with pDUAL-YFH1c-*arf1* and stained with the Golgi-specific stain BODIPY® TR C_5_-ceramide. Arf1-YFP exhibited limited colocalization with BODIPY® TR C_5_-ceramide. B. Wild-type and *gea1+/−* cells transformed with pDUAL-YFH1c-*sac11* were stained with BODIPY® TR C_5_-ceramide. Sac11-YFP localized to the Golgi in *gea1+/−* cells, but not in wild-type cells. C. Wild-type cells transformed with pDUAL-YFH1c-*sac12* and stained with BODIPY® TR C_5_-ceramide showed that sac12-YFP exhibited Golgi localization as expected. Scale bars, 14 µM.
(TIF)
