# Supplementary figures and images for: Haploinsufficiency of the Sec7 Guanine Nucleotide Exchange Factor Gea1 Impairs Septation in Fission Yeast
Source: PLoS One. 2013 Feb 15;8(2):e56807. doi: 10.1371/journal.pone.0056807 (PMC3574105; doi:10.1371/journal.pone.0056807)

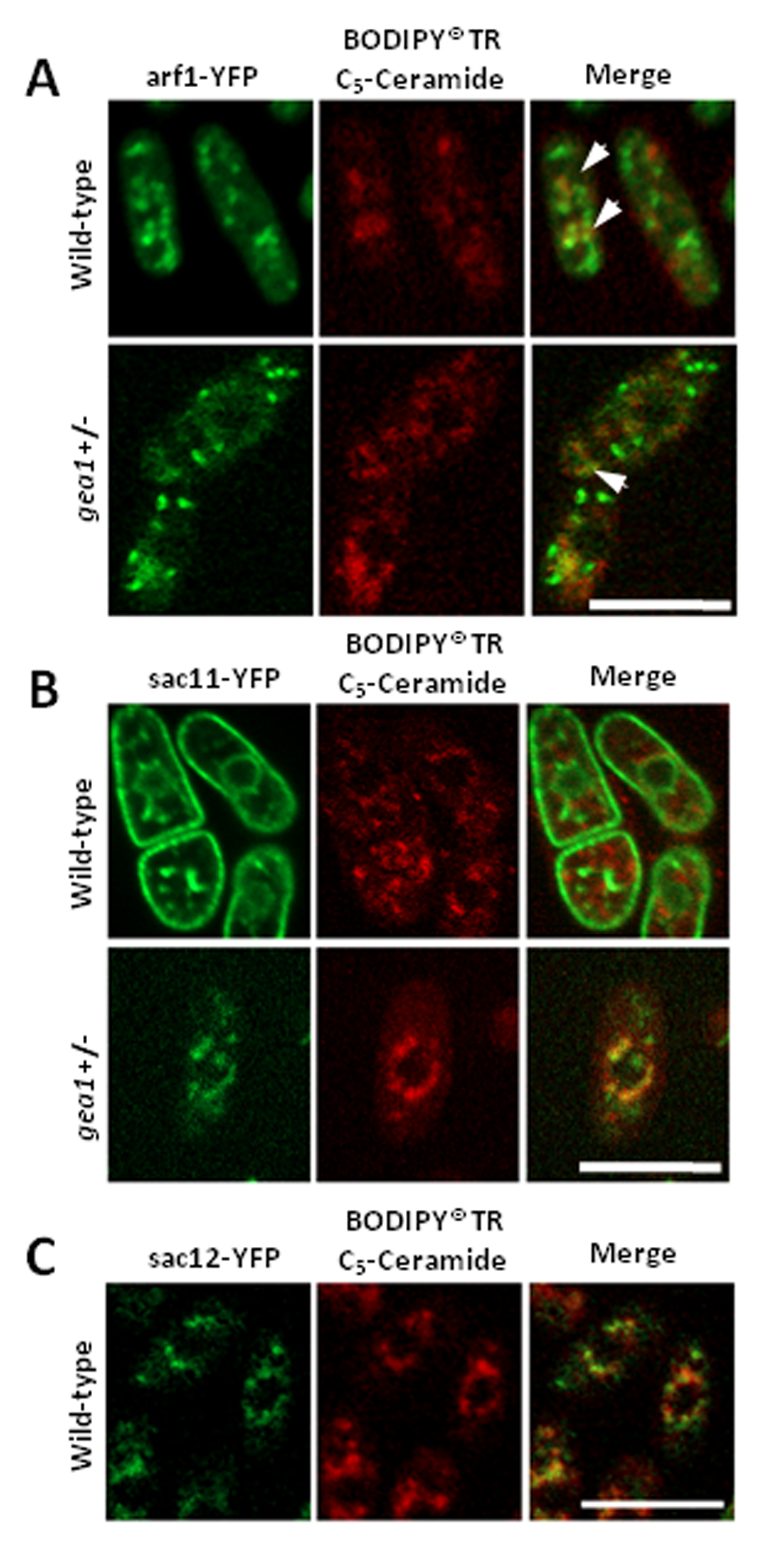

Supplement: Figure S1 — Analysis of Golgi localization. A. Wild-type and gea1+/− cells were transformed with pDUAL-YFH1c-arf1 and stained with the Golgi-specific stain BODIPY® TR C5-ceramide. Arf1-YFP exhibited limited colocalization with BODIPY® TR C5-ceramide. B. Wild-type and gea1+/− cells transformed with pDUAL-YFH1c-sac11 were stained with BODIPY® TR C5-ceramide. Sac11-YFP localized to the Golgi in gea1+/− cells, but not in wild-type cells. C. Wild-type cells transformed with pDUAL-YFH1c-sac12 and stained with BODIPY® TR C5-ceramide showed that sac12-YFP exhibited Golgi localization as expected. Scale bars, 14 µM. (TIF) [file pone.0056807.s001.tif]
